# Supplementary material for: From sequence to enzyme mechanism using multi-label machine learning
Source: BMC Bioinformatics. 2014 May 19;15:150. doi: 10.1186/1471-2105-15-150 (PMC4229970; doi:10.1186/1471-2105-15-150)
Supplement: Additional file 2 — Java code of ml2db. Additional file ml2db_code.tar.gz contains the Java source code to run the multi-label machine learning experiments and save the results to database. The code’s Javadoc is included. [file 1471-2105-15-150-S2.zip › additional file 2/ml2db/ml2db/doc/main/ml2db/al/utility/neighbours/class-use/NeighbourhoodNetwork.html]

Uses of Class main.ml2db.al.utility.neighbours.NeighbourhoodNetwork


JavaScript is disabled on your browser.


- Overview
- Package
- Class
- Use
- Tree
- Deprecated
- Index
- Help

- Prev
- Next

- Frames
- No Frames

- All Classes

## Uses of Class main.ml2db.al.utility.neighbours.NeighbourhoodNetwork

- Packages that use NeighbourhoodNetwork

  | Package | Description |
  | --- | --- |
  |  |  |
  | --- | --- |
  | main.ml2db.al.utility.neighbours |  |
  | main.ml2db.graphviz |  |
  | test.ml2db.al.utility.neighbourhood |  |
- - ### Uses of NeighbourhoodNetwork in main.ml2db.al.utility.neighbours

    Methods in main.ml2db.al.utility.neighbours with parameters of type NeighbourhoodNetwork

    | Modifier and Type | Method and Description |
    | --- | --- |
    |  |  |
    | --- | --- |
    | `static void` | NeighbourhoodExperimentDetailsSerialised.`run(NeighbourhoodNetwork network)` |
  - ### Uses of NeighbourhoodNetwork in main.ml2db.graphviz

    Methods in main.ml2db.graphviz with parameters of type NeighbourhoodNetwork

    | Modifier and Type | Method and Description |
    | --- | --- |
    |  |  |
    | --- | --- |
    | `static void` | GraphVizPrinter.`printNeighboursForGraphviz(java.lang.String filePath, NeighbourhoodNetwork network, boolean inColour)` |
  - ### Uses of NeighbourhoodNetwork in test.ml2db.al.utility.neighbourhood

    Methods in test.ml2db.al.utility.neighbourhood that return NeighbourhoodNetwork

    | Modifier and Type | Method and Description |
    | --- | --- |
    |  |  |
    | --- | --- |
    | `static NeighbourhoodNetwork` | NeighbourhoodNetworkTest.`getNeededNetworkTestset()` |
    | `static NeighbourhoodNetwork` | NeighbourhoodNetworkTest.`getNeededNetworkTrainset()` |

- Overview
- Package
- Class
- Use
- Tree
- Deprecated
- Index
- Help

- Prev
- Next

- Frames
- No Frames

- All Classes
